# The BCL-2 family member BOK promotes *KRAS*-driven lung cancer progression in a p53-dependent manner

**DOI:** 10.1038/s41388-021-02161-1

**Published:** 2022-01-29

**Authors:** Anna-Lena Meinhardt, Enkhtsetseg Munkhbaatar, Ulrike Höckendorf, Michelle Dietzen, Marta Dechant, Martina Anton, Anne Jacob, Katja Steiger, Wilko Weichert, Luka Brcic, Nicholas McGranahan, Caterina Branca, Thomas Kaufmann, Michael A. Dengler, Philipp J. Jost

**Affiliations:** 1 Department of Medicine III, Klinikum rechts der Isar, TUM School of Medicine, Technical University of Munich, Munich, Germany; 2 Cancer Research UK Lung Cancer Center of Excellence, University College London Cancer Institute, Paul O’Gorman Building, London, UK; 3 Cancer Genome Evolution Research Group, University College London Cancer Institute, University College London, London, UK; 4 Cancer Evolution and Genome Instability Laboratory, The Francis Crick Institute, London, UK; 5 Institute of Molecular Immunology and Experimental Oncology, School of Medicine, Technical University of Munich, Munich, Germany; 6 Institute of Pathology, Technical University of Munich, Munich, Germany; 7 German Cancer Consortium (DKTK), Partner Site Munich, Munich, Germany; 8 Diagnostic and Research Institute of Pathology, Diagnostic and Research Center for Molecular BioMedicine, Medical University of Graz, Graz, Austria; 9 Institute of Pharmacology, University of Bern, Bern, Switzerland; 10 Division of Clinical Oncology, Department of Medicine, Medical University of Graz, Graz, Austria; 11 Present Address: Department of Medicine, Rutgers New Jersey Medical School, Newark, NJ USA; 12 Present Address: Department of Surgery, School of Medicine, Technical University Munich, Munich, Germany

**Keywords:** Cancer models, Non-small-cell lung cancer

## Abstract

A variety of cancer entities are driven by *KRAS* mutations, which remain difficult to target clinically. Survival pathways, such as resistance to cell death, may represent a promising treatment approach in *KRAS* mutated cancers. Based on the frequently observed genomic deletions of BCL-2-related ovarian killer (BOK) in cancer patients, we explored the function of BOK in a mutant *Kras*^*G12D*^-driven murine model of lung cancer. Using *Kras*^*G12D*/+^ *Bok*^*−/−*^ mice, we observed an overall tumor-promoting function of BOK in vivo. Specifically, loss of BOK reduced proliferation both in cell lines in vitro as well as in *Kras*^*G12D*^-driven tumor lesions in vivo. During tumor development in vivo, loss of BOK resulted in a lower tumor burden, with fewer, smaller, and less advanced tumors. Using *Kras*^*G12D*/+^ *Tp53*^*Δ/Δ*^ *Bok*^*−/−*^ mice, we identified that this phenotype was entirely dependent on the presence of functional p53. Furthermore, analysis of a human dataset of untreated early-stage lung tumors did not identify any common deletion of the *BOK* locus, independently of the *TP53* status or the histopathological classification. Taken together our data indicate that BOK supports tumor progression in *Kras*-driven lung cancer.

## Introduction

Despite recent therapeutic advances, including the development of immune checkpoint inhibitors and other targeted therapies, lung cancer remains the leading cause of cancer-related deaths worldwide [1]. Lung adenocarcinoma (LUAD) is the most common subtype of lung cancer and is associated with significant rates of morbidity and mortality. About 33% of LUAD cases are associated with activating mutations in *KRAS* [2], which are difficult to target in a clinical setting. Furthermore, 46% of LUAD patients have somatic mutations in the tumor suppressor *TP53*––one of the most common events in cancer [2]. TP53 defends cells from acquired stress by triggering a multitude of anti-proliferative and stress-response signals such as senescence, cell cycle arrest, coordination of DNA damage repair, or apoptosis [3, 4].

Notably, defects in apoptosis can promote tumorigenesis per se through extending the life span of a cell, therefore increasing its chances of acquiring additional cancer-promoting mutations [5]. Furthermore, apoptosis inhibition enables cell resistance to stress caused by oncogenic alterations, nutrient deprivation, DNA damage, or the build-up of toxic components [6]. Under normal conditions, apoptosis is controlled by the BCL-2 protein family [7], which comprises both pro- and anti-apoptotic members. Apoptotic signals result in permeabilization of the mitochondrial outer membrane, caspase activation, and cell death [7]. Interestingly, several cancer entities have altered expression of BCL-2 family members [7–9].

The BCL-2 family member BOK (BCL-2-related ovarian killer) [10] is a multi-BH-domain family member with sequence and structural homology to pro-apoptotic BAX and BAK [11, 12]. BOK localizes primarily to the endoplasmic reticulum (ER) [13], where its activity is negatively regulated by ER-associated degradation [14]. Furthermore, BOK is a critical inducer of apoptosis in response to ER stress [15]. Notably, BOK has also been linked to the pathway of TP53-mediated cellular response to DNA damage, with Zhang et al. showing increased levels of damage in the absence of BOK [16, 17]. Recent data point to a role for BOK also in cell proliferation [18] and Srivastava et al. suggested that BOK interacts with the UMP synthetase enzyme (UMPS), aiding its function in nucleotide synthesis and thus promoting cellular proliferation [19].

While its function and purpose have remained in part elusive, Beroukhim et al. found that the genomic locus containing BOK amongst several other genes is frequently deleted across several cancer entities [20]. These data were initially interpreted as pointing to a previously unknown function of BOK as a tumor suppressor and thus underlining the need for further investigation.

Using an inducible model of *Kras*-driven murine lung cancer, we show that BOK plays a role in promoting tumor development, as *Bok*-proficient mice developed a larger number of more advanced lesions. This effect is mediated, at least in part, by defects in proliferation, and is entirely dependent on p53. Furthermore, we show that the *BOK* locus is not genetically deleted in human lung cancer naïve samples. Collectively, our data point towards a function of BOK in promoting the development of lung adenocarcinomas.

## Results

### *Bok* deletion reduces tumor burden

**Fig. 1:**
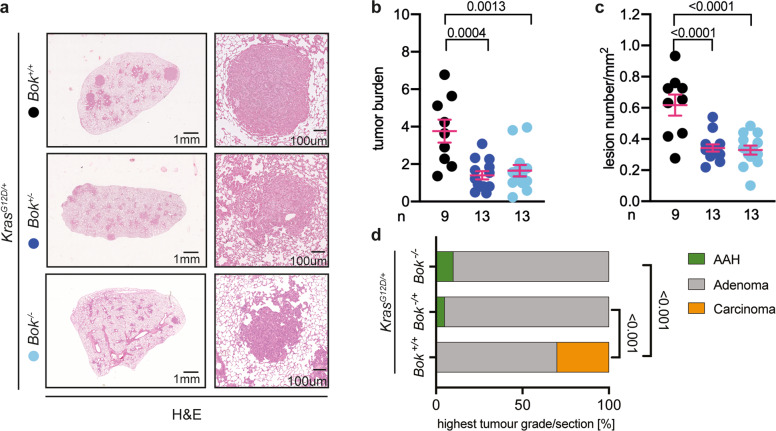
Deletion of *Bok* reduces tumor burden. **a** Representative images of H&E staining of lungs of the indicated genotypes 19 weeks after infection with 5 × 10^6^ PFU of AdenoCre. Panels on the right are higher magnification. Scale bars represent 1 mm or 100 µm, respectively. **b** Quantification of the H&E staining expressed as the ratio between the area of the lesions over the total lung area. Data were analyzed by one-way ANOVA (*p* = 0.0002, *F*(2,32) = 10.93). Reported in the figure as *p* values from the post hoc analysis with Bonferroni correction. **c** Quantification of the H&E staining expressed as number of lesions per mm^2^. Data were analyzed by one-way ANOVA (*p* < 0.0001, *F*(2,32) = 15.51) and are reported in the figure as p values from the post hoc analysis with Bonferroni correction. **d** Percentage of slides that showed at least one lesion histologically graded as indicated by color coding from mice of the indicated genotypes at 19 weeks post-infection. Data were analyzed by chi-square test (*p* < 0.001). For the quantifications reported in panel (**b**–**d**), three sections per animal, separated by 100 µm, were analyzed. n = number of animals. Data in panel (**b**, **c**) are presented as dot plot (every dot represents one experimental animal) and report mean ± SEM. Color code as in panel (**a**).

To analyze the role of BOK in lung cancer, we used mice harboring a *lox-stop-lox-Kras*^*G12D*^ allele, in which the mutated *Kras* is expressed upon AdenoCre viral infection of the lungs [21]. Through this synchronized induction of tumor development, we compared the development of lung cancer between littermate mice with two copies (*Bok*^*+/+*^), one copy (*Bok*^*+/−*^) or none (*Bok*^*−/−*^) of the *Bok* gene. Intriguingly, *Kras*^*G12D/+*^*Bok*^*−/−*^ and *Kras*^*G12D/+*^*Bok*^*+/−*^ mice showed significantly lower tumor burden than the *Bok*-proficient animals (Fig. 1a, b). This was due to a decrease in overall lesion number (Fig. 1c). We then histologically graded the most advanced lesion present in the lungs of experimental mice [21]. Whereas *Bok*^*−/−*^ and *Bok*^*−/+*^ mice more frequently presented with histologically well-differentiated lesions, *Bok*-proficient mice presented with more advanced lesions (Fig. 1d). Notably, most advanced lesions (carcinoma) were only observed in the *Bok*− proficient mice. These data indicate that loss of BOK leads to a lower tumor burden and lesion progression in *Kras*-driven lung cancer.

### *Bok* deletion decreases proliferation

**Fig. 2:**
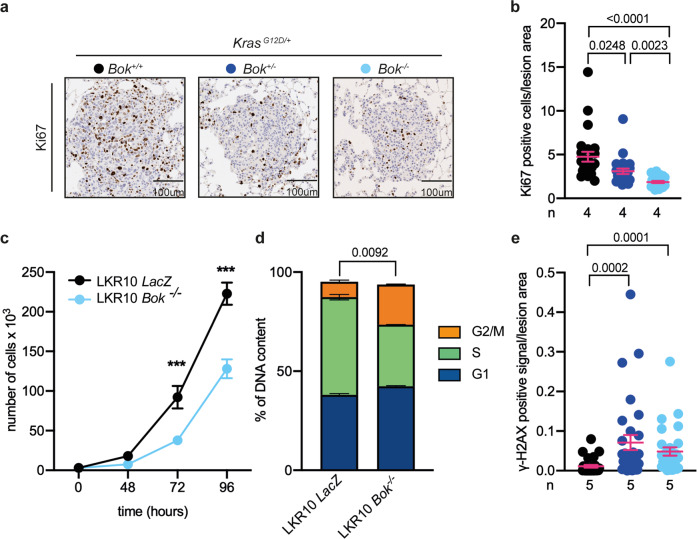
*Bok*-deficiency correlates with a lower proliferation rate and higher levels of DNA damage. **a**, **b** Representative images and quantification of Ki67 staining. Data are reported as number of positive cells normalized to lesion area and analyzed by Kruskal–Wallis test (*p* < 0.0001, *H*(3) = 36.26). In panel (**b**), the *p* values from the post hoc analysis with Dunn´s correction are reported. **c** Live cell numbers evaluated as trypan blue exclusion over time. Data are presented as mean ± SEM and were analyzed by repeated measure two-way ANOVA (time, genotype, and interaction effects, *p* < 0.0001). Post hoc analysis with Bonferroni correction showed a significant difference between the genotypes at 72 h and 96 h (***p < 0.0001). **d** Cell cycle analysis performed with propidium Iodide (PI) staining. Data are presented as mean ± SEM and were analyzed by chi-square test (*p* = 0.0092, chi-square (2) = 9.373). **e** Quantification of γ-H2AX positive staining normalized to lesion area. Data were analyzed by Kruskal–Wallis test (*p* < 0.0001, *H*(3) = 22.34) and are reported in the figure as *p* values from the post hoc analysis with Dunn´s correction. For the quantification in panel (**b**) and (**e**), six lesions per slide/animal were evaluated and data are presented as dot plot and report mean ± SEM. For panel (**c**) and (**d**), each cell line was evaluated in at least three independent experiments.

As the proliferative rate is an important characteristic of malignant lesions and BOK has been shown to affect proliferation [18, 19], we analyzed Ki67 in our setting, a well-established immunohistochemical marker of proliferation. Consistent with a prior publication on hepatocellular carcinoma [18], *Bok*^*+/+*^ lesions showed significantly more Ki67 positive cells than *Bok*^*−/−*^ lesions (Fig. 2a, b). However, we did not observe any differences in the number apoptotic cells in *Bok*^*+/+*^, *Bok*^*+/−*^ or *Bok*^*−/−*^ lesions (Fig. S1).

To further explore the role of BOK, we used the mutant *Kras*-driven mouse lung adenocarcinoma cell line LKR10 [22] to generate *Bok*-deficient cells by CRISPR/Cas9 (LKR10 *Bok*^*−/−*^ and, as control, LKR10 *LacZ*, Fig. S2a). *Bok* knockout had no effects on the protein expression of its close relatives BAK and BAX, or its well-established pro-survival interaction partner MCL-1 [10, 19] (Fig. S2b). As expected, we found that *Bok*-deficient cells proliferate slower than their counterparts (Fig. 2c). Furthermore, cell cycle analysis revealed that *Bok*-deficient cells show longer G2 duration (Fig. 2d), suggesting persistent DNA damage. Following this data, we analyzed the levels of phosphorylated histone γ-H2AX, an indicator of DNA double-strand breaks, in our experimental animals. In line with our hypothesis, we detected higher levels of γ-H2AX in *Bok*-deficient lesions (Fig. 2e). Taken together, these data show that BOK impacts on proliferation as well as on DNA damage repair response, which could explain the decreased tumor burden observed in the absence of BOK.

### Functional *Tp53* mediates the *Bok*− dependent phenotype

**Fig. 3:**
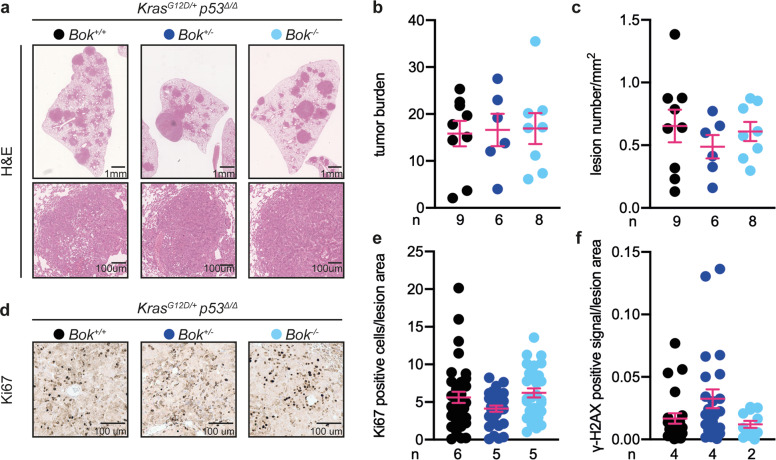
The effect of *Bok* in murine NSCLC is dependent on functional p53. **a** Representative images of H&E staining of lungs of the indicated genotypes 13 weeks after infection with 5 × 10^6^ PFU of AdenoCre. Lower panels are higher magnification of indicated areas. Scale bars represent 1 mm or 100 µm, respectively. **b** Quantification of the H&E staining expressed as the ratio between the area of the lesions over the total lung area. Data were analyzed by one-way ANOVA (*p* = 0.9654, *F*(2,20) = 0.0325). **c** Quantification of the H&E staining expressed as number of lesions per mm^2^. Data were analyzed by one-way ANOVA (*p* = 0.5803, *F*(2,20) = 0.5593). **d**, **e** Representative images, and quantification of Ki67 staining. Data are reported as number of positive cells normalized to lesion area. Data were analyzed by Kruskal–Wallis test (*p* = 0.1013, *H*(3) = 4.579). **f** Quantification of γ-H2AX staining. Data are reported as percentage of positive staining normalized to lesion area. Data were analyzed by Kruskal–Wallis test (*p* = 0.0783, *H*(3) = 5.094). For the quantifications reported in panel (**b**, **c**), three sections per animal, separated by 100 µm, were analyzed. For the quantification in panel (**e**) and (**f**), six lesion per slide/animal were evaluated. n = number of animals. Data in panel (**b**, **c**, **e**, and **f**) are presented as dot plot and report mean ± SEM.

Since alterations in the *TP53* tumor suppressor gene occur in about 50% of NSCLC cases [23], we investigated the contribution of functional p53 signaling to the effects of BOK in lung adenocarcinoma by crossing the *Kras*^*G12D*^ *Bok*^*−/−*^ mice in a *Tp53*^*fl/fl*^ background. We generated mice in which the administration of Cre recombinase allowed for the simultaneous deletion of the tumor suppressor p53 as well as *Kras*^*G12D*^ activation [23]. Notably, analysis of tumor burden showed no differences across genotypes (Fig. 3a–c), suggesting that the function of BOK is dependent on the presence of p53 in this model of tumorigenesis. Accordingly, proliferative rates and DNA damage levels were similar in *Bok*-deficient and -proficient mice (Fig. 3d–f). Knockout of p53 also rescued the defect in cell cycle progression in the *Bok*-deficient mouse lung adenocarcinoma cell line LKR10 (Fig. S3), supporting the findings in the *Kras*^*G12D/+*^ *Tp53*^Δ/Δ^ mouse model.

### *Bok*-deficiency sensitizes NSCLC cells against DNA damaging agents

**Fig. 4:**
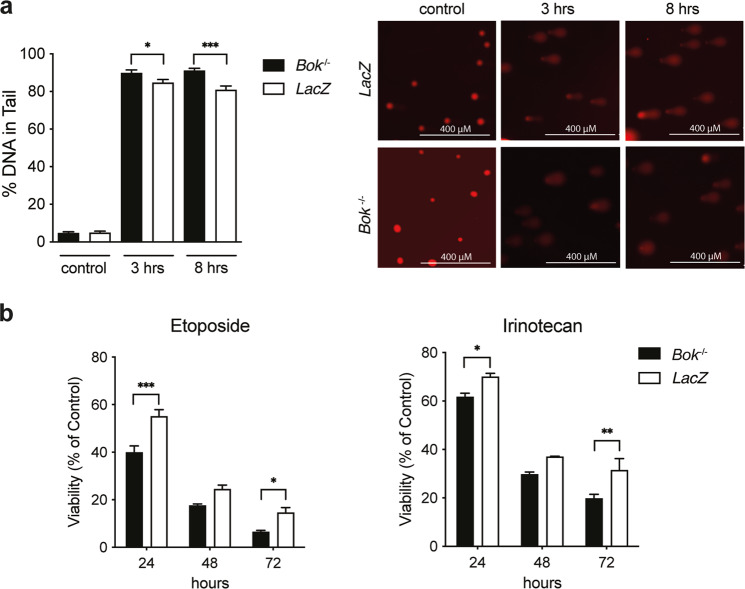
*Bok*-deficiency sensitizes against DNA damaging agents. **a** DNA damage in Etoposide-treated LKR10 *LacZ* and *Bok*^*−/−*^ cells. Cells were treated with 6 µg/ml Etoposide for indicated times and the level of DNA damage (% DNA in tail) analyzed by alkaline comet assay. Percent tail in DNA (% DNA in tail as measured in comet assay) after exposure to Etoposide (left panel). Representative microscopic images of the comet assay (right panel). Data are means ± SEM of at least 25 cells per group analyzed across two slides. Statistical differences at the indicated times were determined by unpaired *t*-test with Welch correction. **b** Cell viability of LKR10 *LacZ* and *Bok*^*−/−*^ cells treated with Etoposide (4 µg/ml) or Irinotecan (80 µM) for indicated times. Data are means ± SEM of three independent experiments. Significant differences from *LacZ* were revealed by two-way ANOVA, Bonferroni’s multiple comparison test (*: *P* < 0.05; **: *P* < 0.01; ***: *P* < 0.001).

As our previous data suggested that *Bok*-deficient lung cancer cells have a defect in DNA repair leading to a p53-dependent arrest in G2 (Figs. 2 and S3), we wanted to determine if *Bok*-deficient cells are thus more sensitive to DNA damaging agents. First, to measure differences in DNA damage and DNA repair capacity in *Bok*-deficient versus *Bok*-proficient lung cancer cells upon induction of DNA damage, we performed comet assays in the LKR10 *LacZ* and *Bok*^−/−^ cells treated with Etoposide. As expected, Etoposide-treated *Bok*-deficient cells had significantly higher levels of DNA damage (% DNA in tail) than the *LacZ* control cells, indicating that *Bok*-deficient cells have a decreased capacity to efficiently repair DNA strand brakes (Fig. 4a). Accordingly, LKR10 *Bok*^−/−^ cells showed significantly increased sensitivity to DNA damaging standard chemotherapeutics such as Etoposide and Irinotecan (Fig. 4b). These results highlight the important role of BOK for DNA repair processes in lung cancer cells.

### The *BOK* locus remains unaffected in human lung cancer patients

**Fig. 5:**
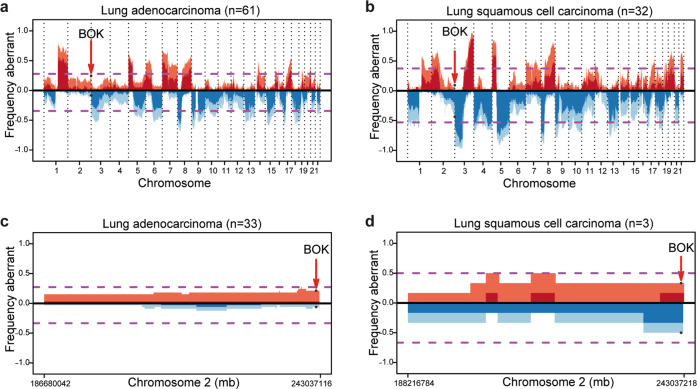
*BOK* is not subjected to copy number variation in lung cancer patients. **a**, **b** Copy number aberrations (CNA) expressed as frequency in TRACERx LUAD (**a**, *n* = 61) and LUSC (**b**, *n* = 32) tumors. **c**, **d** CNA expressed as frequency in TRACERx LUAD grouped for TP53 wildtype (WT) in LUAD (**c**, *n* = 33) and LUSC (**d**, *n* = 3). Shading indicates clonal status. Dashed purple lines represent threshold for frequent gains and losses.

As stated before, *BOK* has been shown to be genomically deleted across several cancer entities [20]. To gain additional information specific to lung cancer, we interrogated an extended dataset of human patients from the TRACERx dataset [24]. The TRACERx prospective study was designed to analyze intra-tumor heterogeneity and the evolutionary trajectory of tumor cell genomes specifically in treatment-naïve lung squamous carcinoma (LUSC) and LUAD patients. Using the TRACERx data, we detected that *BOK* is not frequently deleted in either cohort (Fig. 5a, b). Our data in the mouse models suggest an interplay between BOK and TP53, therefore we re-run the same analysis in the TP53 wild type (WT) subpopulation. Again, both in LUSC and LUAD, we detected no significant *BOK* deletion (Fig. 5c, d).

Taken together, our data suggest that, while BOK plays a role as tumor-promoting factor in lung cancer, and clearly does so in collaboration with TP53, it is not genomically deleted in lung cancer from patients in the TRACERx study.

## Discussion

Despite the fact that the BCL-2 family member BOK was discovered more than 20 years ago, its function still remains ambiguous (reviewed in [25]). Using a constitutional *Bok*^*−/−*^ knockout mouse model, we found that *Bok*-deficiency reduces tumor burden, by decreasing lesion number and histological grading. This phenotype was mainly driven by proliferation deficits both in vivo and in vitro models. Furthermore, these effects were entirely lost in the absence of functional p53, together suggesting a tumor promoting role for BOK.

Due to the structural homology of BOK with the apoptosis effectors BAX and BAK [11, 12] and the aggravated phenotype seen in BAX/BAK/BOK full body triple knockout mice [11], BOK is considered to be a pro-apoptotic member of the BCL-2 family. As such, its deletion should be an advantage in tumor survival and BOK should act as a tumor suppressor. However, recent publications also point towards cell death-independent functions of BOK, including a pro-proliferative role of BOK (first proposed by [26]). *Bok*^*−/−*^ cell lines, including lung cancer cell lines, divided slower [19, 27] and *Bok*^*−/−*^ hepatocellular carcinomas and cell lines showed lower proliferative indexes [18]. In line with these data, we showed that *Bok*-deficient lung cancer lesions have lower Ki67 levels and *Bok*-deficient lung cancer cell line proliferate slower.

It can be argued that apoptosis induction and proliferation inhibition are two sides of the same coin. It is known that apoptosis and cellular proliferation are linked by cell-cycle regulators and signaling pathways, affecting both processes [28]. Under physiologic conditions, apoptosis elicits a signaling cascade inducing cell proliferation in the neighboring cells [29]. Therefore, in our settings, the deletion of *Bok* may decrease proliferation in surrounding cancer cells, by repressing apoptosis in tumor cells.

Furthermore, the proliferative defect seen after downregulation of BOK in human HCT-116 colorectal carcinoma cells is dependent on increased cell cycle arrest, due to the higher levels of the cyclin kinase inhibitors p19^INK4d^ and p21^cip1^ [18]. Along the same line, p53 was found to be upregulated in BOK-deficient cells [19]. These results are in line with our finding, showing that the absence of p53 eliminates any *Bok*-driven differences in vivo and in vitro. It could be argued that, in our mouse models, the differences are driven by a ceiling effect due to the different aggressiveness of the tumor models. However, while this could explain the tumor burden, it could not justify the effects on proliferation and DNA damage.

While Beroukhim et al. found the genomic region containing the *BOK* locus to be deleted in a wide-ranging screen of primary cancer tissue and established cell lines [20], we identified no recurrent deletion of the *BOK* locus in LUAD and LUSC treatment-naïve samples coming from the TRACERx dataset. These results are in line with our in vivo data, in which deletion of *Bok* impaired tumor proliferation. However, our experimental settings do not allow us to exclude a different BOK involvement at later time points. Moravcikova et al. showed detectable BOK mRNA and protein levels in biopsies from early and late-stage lung cancer patients as well as in various NSCLC cell lines, supporting our finding that the BOK gene is not frequently deleted in NSCLC [27]. Interestingly, the same study indicated that BOK may exert a tumor-suppressor-like function at later stages of disease. While our data suggest that *BOK* deletion does not represent a common event in early lung cancer as it interferes with tumor proliferation, it remains to be tested if and at what stage the proliferation defects seen upon loss of BOK are overcome by the numerous other transformative events in the course of tumor development.

Taken together, our data show that BOK promotes proliferation of tumor cells in mutant *Kras*-driven lung cancer and that, in contrast to common opinion, BOK genomic deletion does not represent a recurrent event in lung cancer.

## Materials and methods

### Mouse experiments

The previously described *LSL-Kras*^*G12D/+*^ mice [21] (re-derived on a C57BL/6 J background) were crossed with *Bok*^*−/−*^ mice (generated on a C57BL/6 genetic background [30]) to generate the following genotypes: *LSL-Kras*^*G12D/+*^ *Bok*^*−/−*^, *LSL-Kras*^*G12D/+*^ *Bok*^*+/−*^, and *LSL-Kras*^*G12D/+*^ *Bok*^*+/+*^. In another set of experiments, these mice were backcrossed on a *Tp53*^*fl/fl*^ background [31] to generate: *LSL-Kras*^*G12D/+*^*;Bok*^*−/−*^*;Tp53*^*fl/fl*^, *LSL-Kras*^*G12D/+*^*;Bok*^*−/+*^*;Tp53*^*fl/f*l^, and *LSL-Kras*^*G12D/+*^*;Bok*^*+/+*^*;Tp53*^*fl/fl*^ animals. Mice from the same litters were used as age-matched control animals in all experiments. Mice were grouped according to their genotype. 6-8 week old mice were intranasally infected with 5*10^6^ PFU of adenovirus expressing Cre recombinase, as previously described [32]. Mice were maintained in pathogen-free conditions with free access to food and water. Mice that developed a tumor-unrelated illness during the course of the experiment were euthanized and excluded from the study. All animal experiments were approved by and conduced in accordance with the District Government of Upper Bavaria (AZ: 55.2-1-54-2532-55-12).

### Immunohistochemistry

FFPE lungs from experimental animals were cut into three-step sections at 100 µm intervals to yield slides of 2 µm thickness. One 2 µm slide from each step section was stained with Hematoxylin-Eosin (H&E) for tumor grading and quantitative tumor analysis. Specific immunostainings were performed using the Leica BOND RXm (Leica Biosystems, Nussloch, Germany), using a DAB-based detection system (BOND Dectection System, Leica Biosystem). The following primary antibodies were used: mouse monoclonal Ki-67 (clone B56, cat. #550609, BD Biosciences, NJ, USA), rabbit polyclonal cleaved caspase-3 (Asp175; Cell Signaling Technology, MA, USA), and rabbit monoclonal Phospho-Ser139-Histone H2AX (γ-H2AX clone 20E3, cat. #9718, Cell Signaling Technology, MA, USA). All stained sections were acquired with a SCN400 slide scanner (Leica Biosystem) and analyzed by experimenter blind to the genotype.

For tumor burden analysis, three slides per animal 100 µm apart were evaluated and the lobe and individual lesion areas were defined manually and measured using the Aperio ImageScope software (version 12.4, Leica Biosystems). The total area of the lesions was normalized over the total area of the lung for each slide. The average of three analyzed H&E slides was used for the reported data. For tumor grading, the slides were evaluated by a pathologist blind to the genotype and were classified as hyperplasia, atypical adenomatous hyperplasia (AAH), adenoma, and adenocarcinomas, as previously described [21, 32]. For the analysis of Ki67 and γ-H2AX immunostainings, the six most advanced lesions on each slide by software-based analysis using the Positive Pixel Count v9 algorithm available in the Aperio ImageScope software (version 12.4, Leica Biosystems) and the reported data were normalized to lesion area.

### In vitro experiments

The *Kras*-driven *Tp53* WT mouse lung adenocarcinoma cell line LKR10 [22] was used to target *Bok* using CRISPR/Cas9-mediated genome editing (targeting sequence: 5’-TCCCAGCGTATACCGGAACG-3’). Targeting guides were cloned in the px458 plasmid, which carries also a GFP expressing cassette (pSpCas9(BB)-2A-GFP (PX458) was a gift from Feng Zhang, Addgene plasmid # 48138; http://n2t.net/addgene:48138; RRID:Addgene_48138). LKR10 cells were transfected with the *Bok*-targeting plasmid and the *LacZ* control using Metafectene® (Biontex Laboratories, Germany). GFP positive cells were single sorted using BD FACSAria™ Fusion flow cytometer (BD Biosciences) to generate new cell lines, in which successful deletion of *Bok* was detected by Western blot. The newly generated LKR10 *Bok*^*−/−*^ and LKR10 *LacZ* cells were maintained in RPMI medium 1640 (ThermoFisher Scientific, MA, USA) supplemented with 10% fetal bovine serum (FBS Good Forte, PAN-Biotech, Germany), 1% penicillin/streptomycin, and 2 mM L-Glutamine (ThermoFisher Scientific), at 37 °C in 5% CO_2_ an atmosphere. Cells were routinely checked for mycoplasma contamination. For knockout of *Tp53* in the LKR10 *Bok*^*−/−*^ cells (targeting sequence: 5′-GAAGTCACAGCACATGACGG-3′) an inducible CRISPR/Cas9 system was used as described by Aubrey et al. [33]. For proliferation analysis, 3,000 cells per line were plated in 96 well plates and were counted by Trypan Blue exclusion every 24 h. For cell cycle analysis, cells were collected and fixed in 70% ethanol, followed by Ribonuclease A treatment (RNAse A, ThermoFisher Scientific), and propidium iodide staining (PI, Sigma-Aldrich, MO, USA). Data were acquired by BD FACSCanto (BD Biosciences) and analyzed using the Watson pragmatic algorithm available in the FlowJo software (v. 9.7.6, BD Biosciences) or ModFit LT 5.0 (ModFit LT™). Cell viability in cells treated with chemotherapeutics was determined using the CellTiter-Glo® Luminescent Cell Viability Assay (Promega) according to the manufactures’ instructions. For Western blot analysis, the following antibodies were used: BOK (1:1000, Abcam, ab233072, clone BOK-R1-5-1); BOK (1:200, clone 1–5, provided by TK and already validated in [13]); p53 (1:1000, Cell Signaling, cat #32352, clone #D2H9O); β-actin (1:1000, Cell Signaling, cat #4970, clone #13E5).

### Comet assay

Cells were seeded in 10 cm dishes at a density of 1.5 × 10^6^ the day before the experiment and then treated with Etoposide (6 ug/ml) at 37 °C for the indicated times. The cells were harvested, resuspended in ice-cold PBS at 1.4 × 10^5^, and then prepared using the Trevigen CometAssay Kit and its protocol. The cells were combined with low-melting agarose at a ratio of 1:10 and spread onto the CometSlides. Gelling was achieved at 4 °C in the dark for 10 min and the slides were then placed in 4 °C Lysis Solution overnight. After incubating the slides in alkaline unwinding solution for 20 min at room temperature (200 mM NaOH, 1 mM EDTA), electrophoresis took place at 4 °C and 1 V/cm in alkaline electrophoresis solution (200 mM NaOH, 1 mM EDTA) for 25 min. The slides were dried at 37 °C and DNA was stained in the dark for 15 min using propidium iodide (1 mg/ml stock solution) at a 1:1500 dilution. Slides were analyzed using the OpenComet software tool (https://cometbio.org/index.html).

### TracerX analysis

The TRACERx study was conducted under the favorable opinion from the NRES Committee London––Camden & Islington Research Ethics Committee [24]. Informed consent was obtained from all subjects. *BOK* copy number alteration was evaluated using a similar approach to the one described in [32].

### Statistical analysis

The details of experiments, including sample number, statistical tests used, number of experiments, and dispersion and precision measures, are stated in the figures or figure legends. The data analysis was performed with GraphPad Prism software and in the R statistical environment version ≥3.3.1. The appropriate statistical test was selected depending on data type and distribution. For all statistical tests statistical significance was determined if the *p* value was less than 0.05.

## Supplementary information

Supplemental Information

Supplemental Figure S1

Supplemental Figure S2

Supplemental Figure S3
