# Supplementary material for: The BCL-2 family member BOK promotes KRAS-driven lung cancer progression in a p53-dependent manner
Source: Oncogene. 2022 Jan 29;41(9):1376–82. doi: 10.1038/s41388-021-02161-1 (PMC8881215; doi:10.1038/s41388-021-02161-1)
Supplement: Supplementary file 1 — Supplemental Information [file 41388_2021_2161_MOESM1_ESM.pdf]

Supplementary Information

Supplemental Figure legends

Figure S1: Immunohistochemistry analysis of cleaved caspase-3 in the lungs of *Kras*$^{G12D/+}$ *Bok*$^{-/-}$, *Kras*$^{G12D/+}$*Bok*$^{+/-}$ and *Kras*$^{G12D/+}$*Bok*$^{+/+}$ mice. Quantification of cleaved caspase-3-positive cells in the lungs of indicated genotypes (left panel). Three sections per animal, separated by 100 µm, were analyzed. n=number of animals. Representative images of cleaved caspase-3 staining (right panel).

Figure S2: Evaluation of successful deletion of *Bok* from LKR10 cell line and potential compensatory effects on other BCL-2 family members. (**a**) To evaluate successful deletion of *Bok* in LKR10 cell line, two different antibodies were used. One is commercially available (Abcam 233072) and one was a gift from Thomas Kaufmann (clone 1-5). As shown in the Western blot (uncropped images), *Bok* was successfully deleted in the LKR10 cell line. (**b**) To evaluate possible changes in the expression of other BCL-2 family members upon deletion of *Bok* in LKR10 cells, protein expression of BAK, BAX, MCL-1 and HSP70 were analyzed by Western blot using rabbit polyclonal anti-BAK (#B5897, Sigma); rat monoclonal anti-BAX clone 49F9 (gift from D.C.S. Huang, Walter and Eliza Hall Institute, Melbourne), rabbit polyclonal anti-MCL-1 (#600-401-394S, Rockland), mouse monoclonal anti-HSP70 (clone N6; gift from Walter and Eliza Hall Institute, Melbourne).

Figure S3: Evaluation of the effect of p53 knockout on the cell cycle in LKR10 *Bok*$^{-/-}$ cells. (**a**) Efficacy of p53 KO on LKR10 *Bok*$^{-/-}$ cells in a pooled population. As shown by the Western blot, p53 levels are strongly reduced in the LKR10 *Bok*$^{-/-}$ p53 KO cells compared to the parental cells. (**b**) Knockout of p53 rescues the proliferation defect in *Bok*-deficient cells. Cell cycle distribution in the indicated cell lines was analyzed by propidium Iodide (PI) staining and flow cytometry. Data was analyzed using ModFit LT 5.0 software. Data are presented as mean ± SEM from three independent experiments and were analyzed by chi-square test.
