# Supplementary material for: The BCL-2 family member BOK promotes KRAS-driven lung cancer progression in a p53-dependent manner
Source: Oncogene. 2022 Jan 29;41(9):1376–82. doi: 10.1038/s41388-021-02161-1 (PMC8881215; doi:10.1038/s41388-021-02161-1)

Supplementary Figure 1

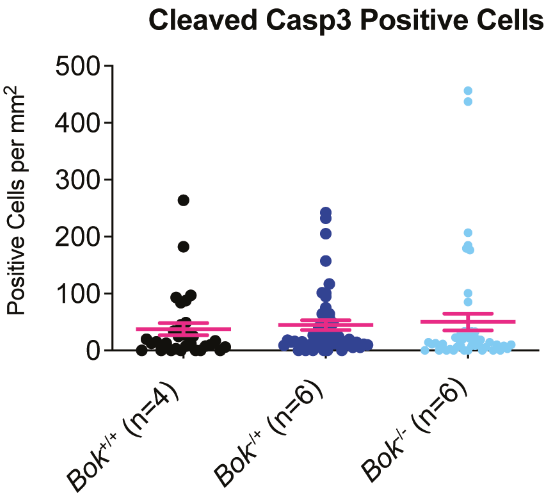

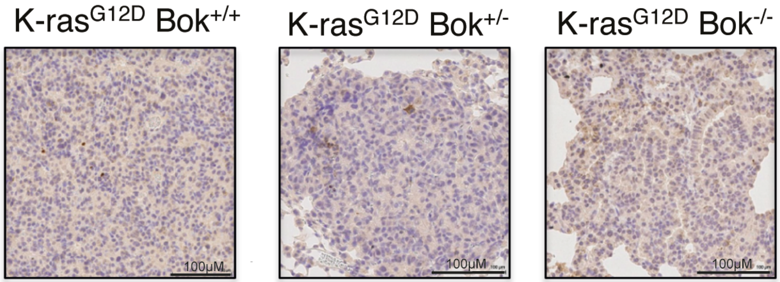

Supplement: Supplementary file 2 — Supplemental Figure S1 [file 41388_2021_2161_MOESM2_ESM.pdf]
