# Supplementary figures and images for: The BCL-2 family member BOK promotes KRAS-driven lung cancer progression in a p53-dependent manner
Source: Oncogene. 2022 Jan 29;41(9):1376–82. doi: 10.1038/s41388-021-02161-1 (PMC8881215; doi:10.1038/s41388-021-02161-1)

**a**

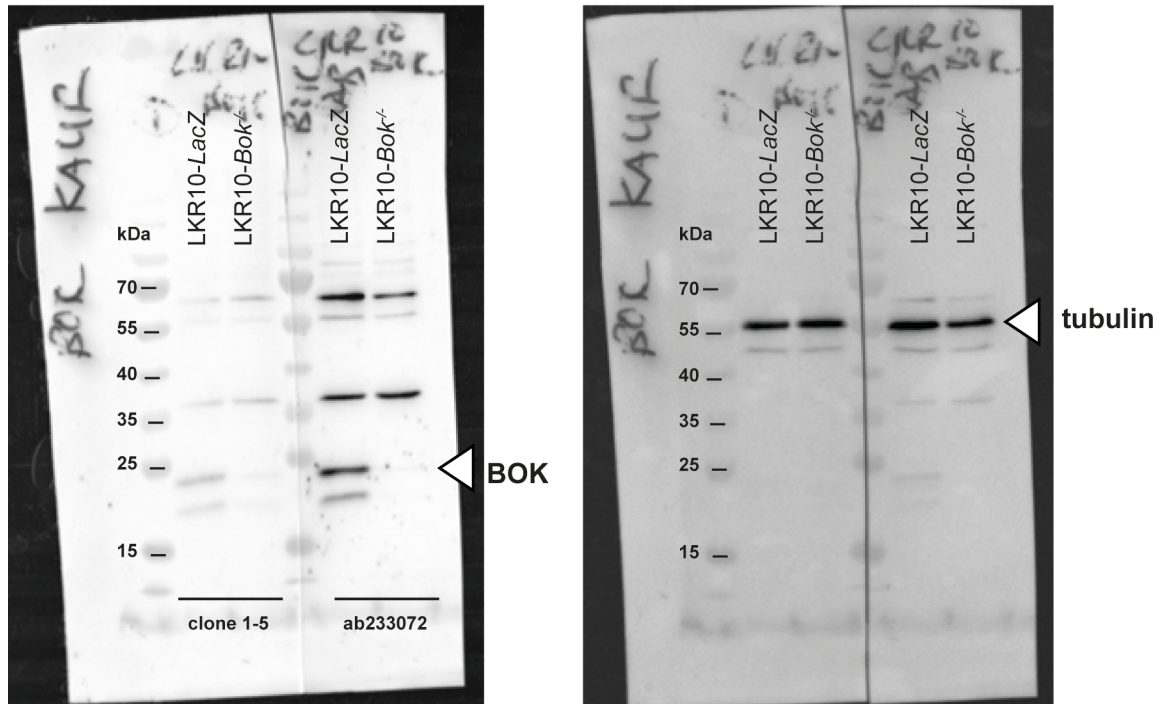

**b**

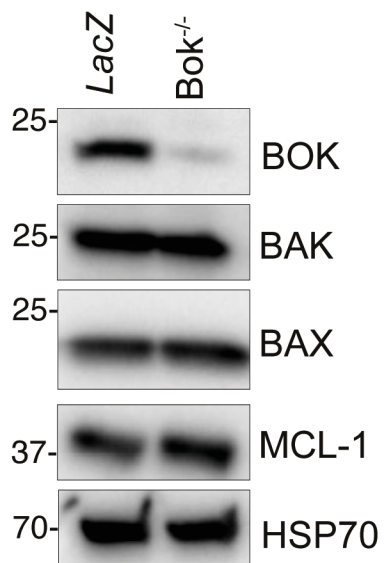

Supplement: Supplementary file 3 — Supplemental Figure S2 [file 41388_2021_2161_MOESM3_ESM.pdf]

## Supplementary Figure 3

**a**

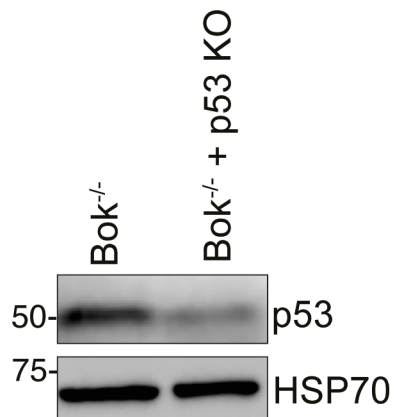

**b**

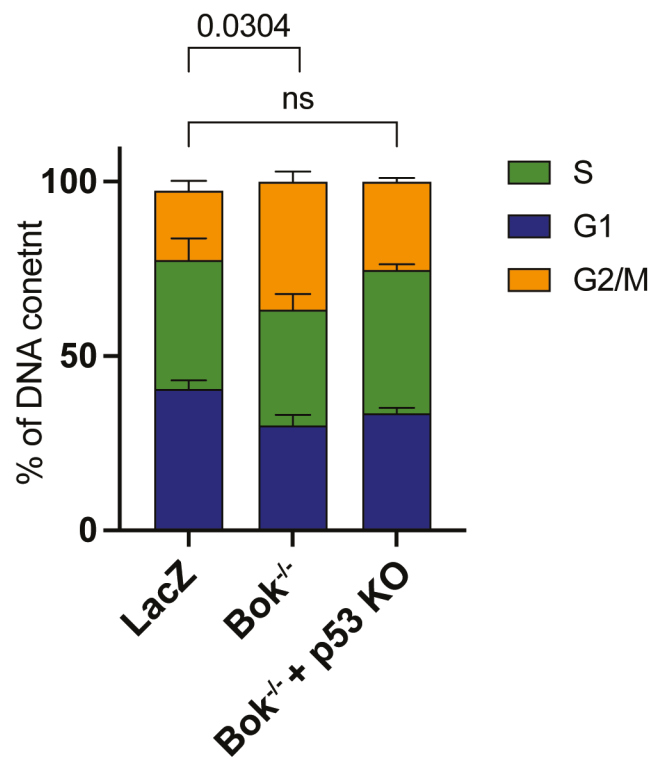

Supplement: Supplementary file 4 — Supplemental Figure S3 [file 41388_2021_2161_MOESM4_ESM.pdf]
